# Understory Dwarf Bamboo Modulates Leaf Litter Decomposition via Interception-Induced Litter Redistribution and Space-Dependent Decomposition Dynamics: A Case Study from Jinfo Mountain, China

**DOI:** 10.3390/plants14203135

**Published:** 2025-10-11

**Authors:** Hai-Yan Song, Feng Qian, Chun-Yan Xia, Hong Xia, Jin-Chun Liu, Wei-Xue Luo, Jian-Ping Tao

**Affiliations:** 1 Key Laboratory of Eco-Environments in Three Gorges Reservoir Region (Ministry of Education), School of Life Sciences, Southwest University, Chongqing 400715, China; haiyansong20@163.com (H.-Y.S.); jinchun@swu.edu.cn (J.-C.L.);; 2 Chongqing Key Laboratory of Plant Ecology and Resources Research in Three Gorges Reservoir Region, School of Life Sciences, Southwest University, Chongqing 400715, China; 3 Chongqing Jinfo Mountain Karst Ecosystem National Observation and Research Station, School of Geographical Sciences, Southwest University, Chongqing 400715, China; 4 Chongqing Jinfo Mountain Field Scientific Observation and Research Station for Kaster Ecosystem, Ministry of Education, School of Geographical Sciences, Southwest University, Chongqing 400715, China; 5 Chongqing Engineering Research Center for Remote Sensing Big Data Application, School of Geographical Sciences, Southwest University, Chongqing 400715, China; 6 Zigong Ecological Environment Monitoring Service Center, Zigong 643101, China

**Keywords:** dwarf bamboo, litter interception, litter decomposition, element release dynamics

## Abstract

Understory vegetation, particularly dwarf bamboo, plays a crucial role in regulating forest nutrient cycles by intercepting litter and altering decomposition processes, yet its overall impacts remain understudied and insufficiently quantified. This study employs a combination of field surveys and decomposition bag experiments to investigate how understory dwarf bamboo (*Fargesia decurvata*) alters the spatial–temporal patterns of leaf litter production and decomposition. We found that the dwarf bamboo intercepted more than 25% of canopy litterfall, altering its spatial distribution and reducing decomposition efficiency in the bamboo crown (BC). Leaf trait-decomposition relationships differed strongly across habitats, being positive for saturated fresh weight (SFW), leaf thickness (LFT), and leaf area (LA) and dry weight (DW) in bamboo habitats but weaker in the bamboo-free habitat (NB). Potassium release was significantly higher in the BC treatment, whereas carbon release showed the opposite trend. In contrast, nitrogen and phosphorus exhibited net enrichment across all treatments, with phosphorus enrichment being slower in BC than in bamboo-covered ground surface (BG) and NB. Our results demonstrate that the understory dwarf bamboo reshapes the spatial distribution of litter and nutrient release dynamics during decomposition, resulting in element-specific nutrient release patterns. These findings provide mechanistic insights into how understory dwarf bamboo mediates nutrient cycling dynamics in forest communities.

## 1. Introduction

Leaf litter, the post-senescence product of plant growth in forest ecosystems, serves as a critical conduit for nutrient cycling and energy transfer, forming the primary input to soil nutrient pools [1]. Understory vegetation, as a secondary ecological interface within forest communities [2], plays a crucial role in understory ecological processes and profoundly alters the nutrient cycling and accumulation [3]. Notably, a substantial proportion of canopy litterfall is intercepted by understory vegetation before reaching the forest floor in mountain forests [4], altering both vertical nutrient flux efficiency and horizontal litter distribution patterns [5]. This interception-mediated redistribution may fundamentally reshape biogeochemical cycling processes and forest ecosystem functions.

However, current research frameworks often overlook understory interception mechanisms, predominantly assuming direct litter deposition at the soil interface to initiate decomposition [6,7]. This conceptual gap becomes increasingly consequential given the climate change-driven expansion of understory vegetation, which may amplify interception capacities. Dwarf bamboos (e.g., *Fargesia decurvata*), prevalent in subtropical forests across East Asia and South America [8,9], exemplify this phenomenon through their morphological adaptations: clustered growth patterns, dense branching, large leaf inclination angles, and overlapping leaves create efficient litter-intercepting funnel structures.

In recent decades, global warming and human disturbance have significantly increased the distribution and stem density of dwarf bamboo [9,10]. Their exceptionally high leaf area index (e.g., some bamboo species LAI = 8) creates near-impenetrable shrub canopies, reducing photosynthetic photon flux density (PPFD) at ground level by 92% [11,12]. This structural configuration markedly increases the probability of litter accumulation within dwarf bamboo crowns. This underscores that the interception effect of dwarf bamboo on canopy litterfall cannot be overlooked [8,9], as it may disrupt decomposition processes, nutrient cycling, and overall forest ecosystem functioning. Although a small but growing body of research has recognized litterfall interception by understory vegetation [5,11], such as Dearden and Wardle’s findings that the dense fern understory exerts significant interception and decomposition effects in temperate rainforests of southern New Zealand [13]. Nevertheless, critical knowledge gaps persist regarding the decomposition patterns of intercepted litter in specialized plant communities, such as those dominated by extremely dense understory vegetation. Previous studies have found that dense fern understories can intercept a substantial amount of canopy litter (with an interception rate of 10%) [13]. In comparison, understory dwarf bamboo, with its dense foliage and lignified structure, is likely to exhibit even higher interception efficiency. However, quantitative studies on dense dwarf bamboo understories remain scarce, despite evidence that large bamboo species (e.g., moso bamboo) strongly influence nutrient cycling and decomposition dynamics [14]. Therefore, elucidating the spatio-temporal distribution patterns and driving factors of dwarf bamboo-mediated litter decomposition is crucial for understanding the functional role of understory vegetation in forest nutrient cycling.

We designed this study to: (1) quantify the capacity of the dwarf bamboo *F. decurvata* to intercept canopy-derived litter using continuous field monitoring; (2) compare decomposition dynamics and element-release rates of litter from 18 dominant tree species—spanning a range of leaf functional traits (e.g., leaf area, thickness)—across three microhabitats: bamboo crown (BC), bamboo-covered ground (BG), and non-bamboo ground (NB); and (3) identify the primary drivers and mechanisms responsible for habitat-specific differences in decomposition and nutrient release. To achieve these objectives, we continuously monitored canopy litterfall to estimate interception by *F. decurvata*, and conducted in situ litterbag decomposition experiments using materials from 18 species to evaluate mass-loss and nutrient release over time in the three microhabitats. Our findings would enable us to characterize the decomposition patterns of intercepted litter within bamboo canopies and clarify the primary drivers and mechanisms governing litter decomposition in both canopy and ground habitats in subtropical montane secondary forests.

## 2. Materials and Methods

### 2.1. Study Site

**Figure 1:**
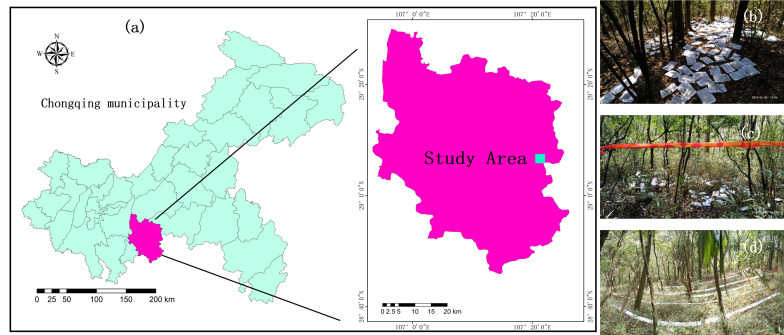
Schematic diagram of the location of the study area in Chongqing and the layout of litter decomposition bags in the field. (**a**) Schematic diagram of the study area location. (**b**) Litterbags were placed on the ground surface in the absence of bamboo for decomposition (NB habitat). (**c**) Litterbags were positioned on the bamboo-covered ground surface (BG habitat). (**d**) Litterbags were suspended within the dwarf bamboo crown (BC habitat).

The experimental site was situated on Jinfo Mountain (28°57′ N, 107°11′ E), Nanchuan District, Chongqing, China (Figure 1a). The mountain exhibits distinct vertical soil zonation, with soil types transitioning sequentially from yellow soil at the foothills to dark brown soil, yellow-brown soil, and brown soil at the summit. Local climate is humid-subtropical monsoonal, with yearly mean air temperature of 8.2 °C and total precipitation of about 1434 mm. The forest is characterized as an evergreen-deciduous broadleaf mixed forest, with the tree layer dominated by species such as *Symplocos sumuntia*, *Cinnamomum wilsonii*, and *Symplocos setchuensis* (Detailed information on the species is provided in Table S1). We established a 1-hectare fixed plot in the study area featuring an even tree canopy, encompassing both bamboo-understory and non-understory areas. The understory shrub layer is dominated by *F. decurvata* (mean height: 0.8 m; crown size: 0.4 m × 0.4 m; density: 76.29 ± 6.51 culms/m^2^) [12]. In recent years, dwarf bamboo has rapidly expanded into previously bamboo-free areas, with increasing density in existing bamboo-dominated regions.

### 2.2. Litterfall Collection and Litter Interception Evaluation

In the bamboo-covered area of the fixed observation field, 18 pairs of litter collection frames were established, with each pair consisting of one ground frame (beneath the bamboo crown) and one canopy frame (above the bamboo crown) deployed adjacently. The collection frames were constructed from nylon nets with a 2 mm mesh size. The cross-sectional area of each collection frame was 1 m × 1 m, and each group of collection frames was at least 5 m apart.

The litter in the litter collection frame was collected monthly, and the litter of bamboo in the ground collection frame was sorted out after each collection. All litter was transported to the laboratory, oven-dried at 65 °C for 72 h, and weighed to determine the dry mass. The litter mass of intercepted by the dwarf bamboo understory (*m_i_*, kg ha^−1^ y^−1^) and the interception rate (*R_i_*, %) were calculated as follows:

(1) $$m_{i}=m_{1}-m_{2}-m_{bamboo}$$

(2) $$R_{i}=\frac{m_{i}}{m_{1}}\times 100\%$$

where *m*_1_ represents the litter mass above the dwarf bamboo canopy (kg ha^−1^ y^−1^), *m*_2_ is the litter on the ground beneath the bamboo, and *m_bamboo_* is the litter mass of the dwarf bamboo. Litterfall for evaluation the litter interception effect was conducted from August 2012 to October 2016 at monthly intervals, except for the snow-covered period of January–March each year.

### 2.3. Litter Decomposition Experiment Design

#### 2.3.1. Litter Decomposition Plot Setup

Two adjacent plots with similar canopy structures were selected within the fixed observation field to conduct the decomposition bag experiment: one with dwarf bamboo (*F. decurvata*) understory cover and one free of dwarf bamboo, each containing three replicates. In the non-bamboo plot, litterbags were placed on the ground surface for decomposition (non-bamboo habitat, NB). In the bamboo-covered plot, litterbags were deployed in two distinct configurations: (1) positioned on the ground surface (bamboo-ground habitat, BG) and (2) suspended within the dwarf bamboo crown (bamboo-canopy habitat, BC). The litterbags are collected in quarterly batches (4 times in total): July 2019 (1 st batch), October 2019 (2 nd batch), January 2020 (3 rd batch), and April 2020 (4 th batch).

#### 2.3.2. Species Selection and Experimental Operation

Litter from 16 dominant wood species and two dwarf bamboo species were selected for decomposition experiments, representing a wide range of leaf traits (leaf thickness: 0.112–2.839 mm; leaf area: 0.798–99.720 cm^2^), encompassing the diversity of leaf morphologies in the forest community (Table S2). Senescent leaves were collected and acted as leaf litter for trait measurements. Leaf thickness (LFT, mm/single leaf) was measured using a thickness gauge (AWT-CHY01), and leaf area (LA, cm^2^/single leaf) was determined using a scanner (EPSON V19). Leaf saturated fresh weight (SFW, g) and dry weight (DW, g) were weighed using an electronic balance. We calculated the leaf dry matter content (ratio of leaf dry weight to saturated fresh weight, LDM, g/g), specific leaf area (ratio of leaf area to dry weight, SLA, cm^2^/g) and leaf tissue density (ratio of leaf dry weight to leaf volume, LTD, g/cm^3^).

Air-dried leaves (approximately 5 g per bag) were enclosed in nylon litterbags (20 cm × 15 cm; aperture 2 mm on the far ground; aperture 1 mm on the near ground), with about 5 g samples per bag. The decomposition bags were deployed across three habitats (NB, BG, BC), with those in the BC habitat secured to the bamboo canopy using wire (Figure 1a–d).

#### 2.3.3. Decomposition Index

Residual mass was determined by gently brushing retrieved samples to remove adhered sediments, followed by oven-drying at 60 °C for 72 h to determine mass loss. Total carbon (C) and nitrogen (N) concentrations were measured using an elemental analyzer (Vario EL III, Elementar), with C/N ratios calculated directly. Phosphorus (P) and potassium (K) contents were determined via inductively coupled plasma optical emission spectrometry (ICP-OES; iCAP 7400, Thermo Scientific, Waltham, MA, USA), enabling calculation of C/P and N/P ratios.

Dry matter loss rate (*D_t_*, %) and nutrient release rate (*E_i_*, %) of litter were calculated using the following formulas [15]:

(3) $$D_{t}=\frac{M_{0}-M_{t}}{M_{0}}\times 100\%$$

(4) $$E_{i}=\frac{e_{0}-e_{i}}{e_{0}}\times 100\%$$

where *M*_0_ and *M_t_* represent the initial mass and the residual mass of the sample at time *t*, respectively, and *e*_0_ and *e_i_* represent the initial nutrient content and the nutrient content of the sample at time *i*. *e_i_* > 0 indicates net nutrient release, whereas *e_i_* < 0 signifies net nutrient accumulation.

Decomposition fitting was performed using the Olson exponential decay model [16]:

(5) $$y=ae^{-kt}$$

where *y* is the percentage of mass remaining (%), *k* (year^−1^) is the decomposition constant, *a* is the fitting parameter, and *t* is the decomposition time. The model is expressed as:

(6) $$M_{t}=M_{0}e^{-kt}$$

(7) $$k=\frac{ln(\frac{w_{0}}{w_{t}})}{t}$$

(8) $$t_{0.95}=\frac{ln0.05}{k}$$

where *t*_0.95_ represents the time required for 95% decomposition.

### 2.4. Statistical Analysis

To evaluate species and habitat effects, repeated-measures ANOVA was applied to litter decay rates and residual C, N, P and K contents. One-way ANOVA plus Duncan’s multiple-range comparison (*p* = 0.05) was applied per collection interval to test habitat effects on decay and nutrient release. Exponential regression was used to model changes in mass loss and nutrient release rates over the decomposition period in each habitat. Repeated random pairwise sampling of litter decomposition and redundancy analysis (RDA) were performed using R. Other Statistical analyses were performed using SPSS 24.0 and Excel 2010. Figures were created with Origin 2024 and R 4.5.0.

## 3. Result

### 3.1. Interception of Canopy Litterfall by Understory Dwarf Bamboo

**Figure 2:**
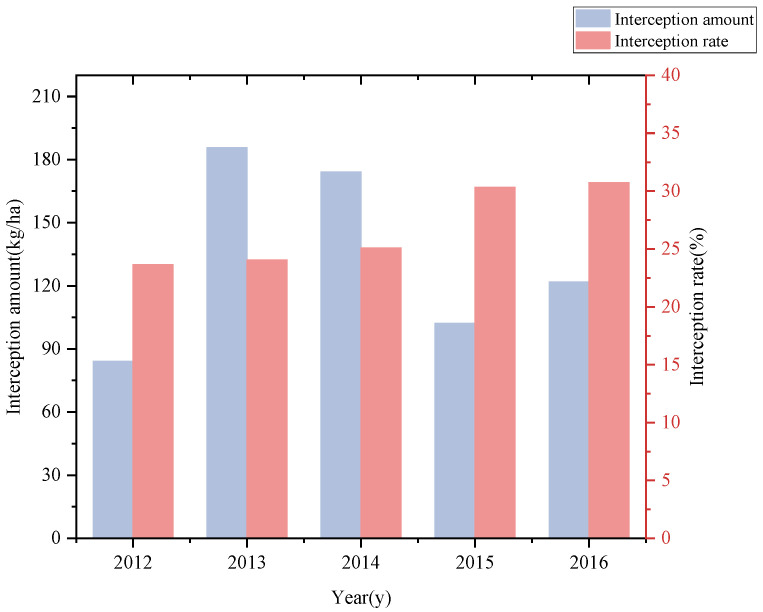
The interception effect of dwarf bamboo on litterfall.

Understory dwarf bamboo had a strong interception effect on tree litterfall (Figure 2), with an average annual interception of litter as high as 133.61 kg/ha. In 2015 and 2016, the interception rate of litter exceeded 30%, and the annual average interception rate of canopy litterfall accounted for over 25% of the total forest litter production.

### 3.2. Leaf Litter Decomposition Dynamics and Decomposition Rates in Different Habitats

**Figure 3:**
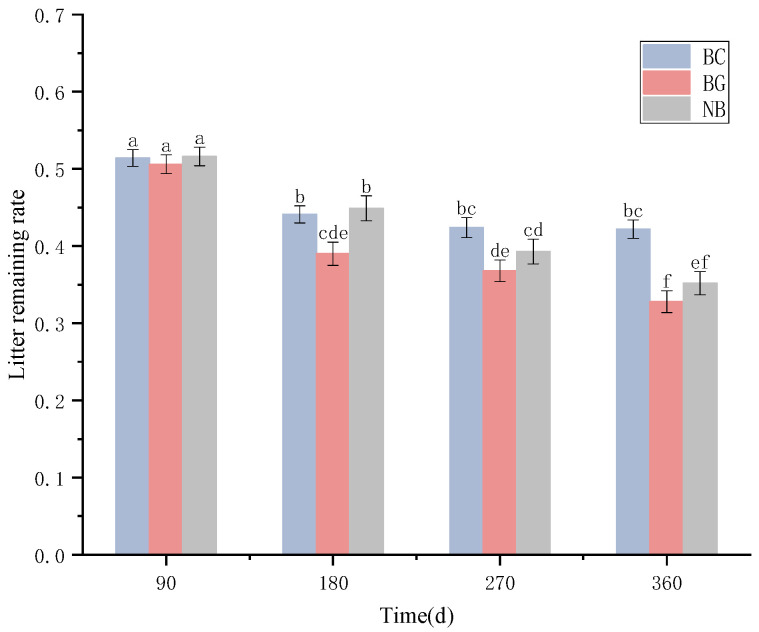
Litter remaining rate in three habitats. Note: Different lowercase letters indicate significant differences in community-level characteristics among different decomposition habitats (*p* < 0.05). BC represents the bamboo canopy habitat, BG represents the bamboo-covered ground, and NB represents the bamboo-free ground. Bars are means ± SE.

As shown in Figure 3, the remaining litter rate in the BC was significantly higher than in the BG and NB habitats throughout the decomposition process. After 90 days, no significant differences in litter remaining rate were detected among the habitats (*p* > 0.05). In the BC habitat, the remaining litter rate exhibited no significant difference between 180 and 270 days (*p* > 0.05), but decreased significantly by 360 days (*p* < 0.05). In contrast, the NB habitat exhibited a consistent linear decrease in litter remaining rate across all four sampling periods, with statistically significant reductions (*p* < 0.05). After one year of decomposition, no significant difference was observed in the remaining litter rate between the BC and NB habitats (*p* > 0.05).

**Figure 4:**
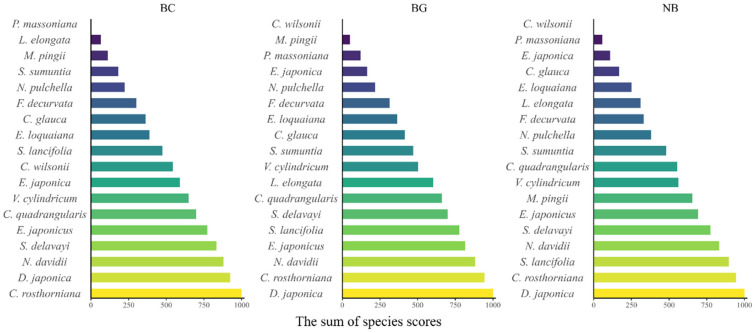
Repeated random pairwise sampling of litter decomposition rate *k*. Note: Species with a score less than 10 are represented in white. BC represents the bamboo canopy habitat, BG represents the bamboo-covered ground, and NB represents the bamboo-free ground.

At the species level (mean of 18 species, Table S3), decomposition rates of litter were significantly affected by the three different decomposition habitats. The BG habitat exhibited the highest decomposition efficiency, with shorter half-life (t_0.5_) and turnover time, followed by the NB habitat. The BC habitat showed the lowest decomposition efficiency, with a t_0.5_ of 2.46 years, a t_0.95_ of 10.65 years, and a decomposition constant (*k*) of 0.28. The repeated random pairwise sampling of litter decomposition rate (*k*) (Figure 4) revealed significant differences in decomposition characteristics among species, with some exhibiting markedly varied decomposition rates across different habitats. Specifically, *Pinus massoniana*, *Symplocos lancifolia*, *Symplocos sumuntia*, and *Litsea elongata* exhibited the slowest decomposition rates and longest turnover times in the BC habitat, while demonstrating comparatively higher decomposition rates in the BG and NB habitats. In contrast, *Cinnamomum wilsonii* and *E. japonicus* decomposed most rapidly in the BC habitat but showed slower decomposition in ground habitats (BG and NB). Meanwhile, *Machilus pingii* and *Neolitsea pulchella* exhibited accelerated decomposition in the bamboo-associated habitats (BG and BC) but significantly slower rates in the bamboo-free habitat (NB). The remaining species displayed similar rankings in both *k* value scores across the three habitats (Figure 4).

### 3.3. Litter Leaves Element Release Dynamics in Different Habitats

After one year of decomposition, carbon (C) and potassium (K) exhibited net release in all three habitats, while nitrogen (N) and phosphorus (P) showed net enrichment. Elemental release patterns varied significantly across decomposition habitats (Table 1). In the BC habitat, the release rate of potassium was significantly higher than that in the BG and NB habitats. The release rate of C was highest in the BG habitat and increased over time. Nitrogen followed a pattern of initial enrichment followed by release, with no significant differences among habitats. Phosphorus showed a similar pattern, with greater enrichment in the BG and NB habitats than in the BC habitat. After 90 days, P enrichment in the BG and NB habitats was approximately 3.3 times higher than in the BC habitat, with this difference diminishing as decomposition progressed.

### 3.4. Factors Influencing Litter Decomposition Across Different Habitats

Litter decomposition was significantly influenced by leaf type, decomposition habitat, decomposition time, and their interactions (Table 2). Specifically, these factors significantly affected residual litter mass, C content, C/N ratio, and N/P ratio (*p* < 0.001). Decomposition time strongly influenced residual litter mass (*F* = 12078, *p* < 0.001) and the elemental release process (*p* < 0.05). Habitat had no significant effect on N and P release (*p* > 0.05), and the interactions among species, habitat, and time did not significantly influence the release of P and K (*p* > 0.05).

The redundancy analysis (RDA) revealed pronounced differences in litter decomposition rates among the three habitats, with RDA1 and RDA2 explaining 43.9% and 26.2% of the variance, respectively. In the BC habitat, decomposition rates were positively associated with leaf area (LA), leaf thickness (LFT), leaf dry weight (DW), and structural traits (SFW). A similar but weaker association was observed in the BG habitat. By contrast, in the NB habitat, species with higher specific leaf area (SLA) decomposed more rapidly, reflecting a decomposition pattern distinct from that in bamboo-dominated habitats. Across all habitats, leaf tissue density (LTD) and leaf dry matter content (LDM) were consistently negatively correlated with decomposition rates.

## 4. Discussion

### 4.1. Interception Effect of Dwarf Bamboo on Litterfall of Forest Canopy

In montane forest ecosystems, canopy-derived litter falls directly to the soil surface via gravity, undergoing leaching and decomposer-mediated fragmentation to release nutrients, thereby sustaining ecosystem nutrient and elemental cycling [17,18]. However, dense understory vegetation, such as dwarf bamboo, disrupts this process by intercepting a significant proportion of litterfall, altering its spatial distribution, and potentially modifying decomposition trajectories and nutrient cycling dynamics [4]. Interannual climate variability, particularly drought events and rising temperatures, induces mast-year fluctuations in canopy litterfall production. This, in turn, drives corresponding interannual variations in the total litter mass intercepted by the understory dwarf bamboo [19,20]. Our study quantified the interception capacity of dense dwarf bamboo, revealing that over 25% of the annual canopy litterfall is retained within bamboo crowns, with per-unit-area litter interception exceeding expectations. This efficient litter interception effect may be attributed to multiple factors. Firstly, the growth form and structure of dwarf bamboo enable it to intercept litter effectively. The dense bamboo culms and high stem density form a physical barrier that prevents litter from falling directly and spreading. The configuration of bamboo branches forming a sharp angle with the bamboo culms, combined with the adjacent dense branches, creates a funnel-shaped sieve. Furthermore, the thick leaf layer, characterized by a high leaf area index (LAI) and the large leaf area of bamboo, can easily catch the falling litter [21,22]. During the investigation period, the individual density of the understory dwarf bamboo increased annually, which may also explain the year-by-year rise in its interception rate. Additionally, litter morphology also influences interception outcomes, with larger and lighter litter more likely to be intercepted by dwarf bamboo [23,24], while smaller, heavier litter may more easily penetrate the bamboo canopy to reach the ground surface [25]. These findings demonstrate that dwarf bamboo understories intercept over one-quarter of canopy-derived litter, highlighting the important role of understory vegetation in regulating forest nutrient cycles.

### 4.2. Effect of Understory Dwarf Bamboo on Litter Decomposition

Dwarf bamboo are rapidly expanding across East and Southeast Asian forests [9,26], forming monodominant shrub layers that intercept a substantial fraction of canopy litter [27]. As evidenced in this study, such a significant proportion of forest litter could not reach the forest floor, but rather remain suspended above the ground in the bamboo canopy, where it will complete the decomposition process independently of the soil environment [28]. Our findings revealed that after one year of decomposition, litter held by bamboo crown habitat retained 60% residual mass—significantly higher than that decomposed in bamboo ground habitats. Litter held by BC exhibited a longer half-decay period 0.5 years than BG habitats, indicating pronounced decomposition hysteresis caused by interception effect of dwarf bamboo at the community level. This delay may reflect microhabitat modifications at the soil surface: greater moisture retention through reduced evaporation [29], enhanced thermal buffering and accumulation in surface soils [30], and the consequent stimulation of microbial activity [21]. Furthermore, ground habitats support richer soil faunal communities with higher population densities and taxonomic diversity [31]. These soil faunal enhance decomposition through bioturbation—increasing litter-soil contact interfaces, oxygen diffusion, and water infiltration—while directly fragmenting litter into smaller, more bioavailable particles [32,33]. Collectively, such synergistic abiotic-biotic drivers underpin accelerated decomposition in BG habitats.

In contrast, the intercepted litter is suspended in the bamboo canopy, far away from the ground, resulting in a decomposition environment that is highly susceptible to strong changes due to fluctuations in the air environment [34]. This imposes strong environmental filtering on both decomposer taxa and their activity, which is not conducive to the construction of decomposer communities, thus leading to the retardation effect and inefficiency phenomenon of the decomposition of litter intercepted by the bamboo canopy.

In addition, we observed that BC-retained litter fragments progressively diminish in size and mass under natural conditions (non-bag confined). Wind, precipitation, and gravity eventually transport these reduced particulates to BG habitats for subsequent decomposition stages [34]. This decomposition pathway of spatial–temporal decoupling—involving aerial decomposition followed by terrestrial decomposition—may fundamentally reorganize forest nutrient cycling. Therefore, investigating this process could advance understanding of complex forest ecosystem functioning.

### 4.3. Litter Decomposition Dynamics and Nutrient Release

Our study revealed that accumulated decomposed litter mass increased over time while the litter decomposition rate declined. This pattern aligned with earlier work that attributed the initial mass loss of litter to the leaching of soluble carbon and the preferential microbial utilization of labile compounds [35]. During later decomposition stages, the slower litter decomposition rate was more likely to be attributed to the functional traits of litter, such as lignin, phenols, and high C/N, which made litter more difficult for microorganisms to use and resulted in slower decomposition rates [36]. In other words, it can be attributed to the fast decomposition rate of litter was mostly driven by biotic factors in the initial decomposition stage, whereas in the later stages, driven by litter’s slowly degradable compounds and environmental constraints [35].

During litter decomposition, nutrient elements were translocated, exhibiting distinct migration patterns across habitats and over time. Overall, prolonged decomposition induced net release of carbon and potassium from litter [37]. Carbon release efficiency showed a temporal decline, attributable to concentration-dependent dilution effects caused by cumulative carbon losses during advanced decomposition stages [37]. Notably, potassium exhibited pronounced net release, with significantly higher liberation rates in bamboo canopy habitats compared to bamboo-covered ground and non-bamboo forest floor environments. The significantly higher release rate of K in bamboo canopy habitats can be attributed to its high solubility and rapid leaching under canopy interception and rainfall exposure [38]. Furthermore, reduced humus thickness and sparse fine-root networks in non-bamboo areas likely indirectly influence K leaching processes through altered soil physicochemical properties [38].

The net enrichment of N and P in decomposing litter materials may result from atmospheric deposition and canopy throughfall inputs, as well as microbial immobilization within the litter matrix [39,40,41]. Notably, after three months of decomposition, phosphorus (P) concentrations in bamboo ground (BG) and non-bamboo floor habitats were approximately 3.3 times higher than those in bamboo canopy habitats. The mineralization of phosphorus (P) in litter lags behind carbon loss. As microorganisms rapidly respire organic carbon as CO_2_, phosphorus—mineralizing more slowly—accumulates in the remaining litter, raising its concentration relative to dry mass. This enrichment is pronounced in ground habitats (BG and NB), where accelerated dry matter loss amplifies the effect, whereas the limited decomposition in bamboo canopy (BC) results in weaker enrichment [42]. This disparity also likely reflects decomposer demand for P in cellular structure synthesis [43], indirectly suggesting richer microbial communities in ground habitats compared to canopy environments. However, such habitat-driven differences diminished with prolonged decomposition. Unexpectedly, habitat-specific trends in C/N and N/P ratios remained obscure, highlighting the complex stoichiometric regulation during elemental cycling.

### 4.4. Interspecific Variation in Litter Decomposition

**Figure 5:**
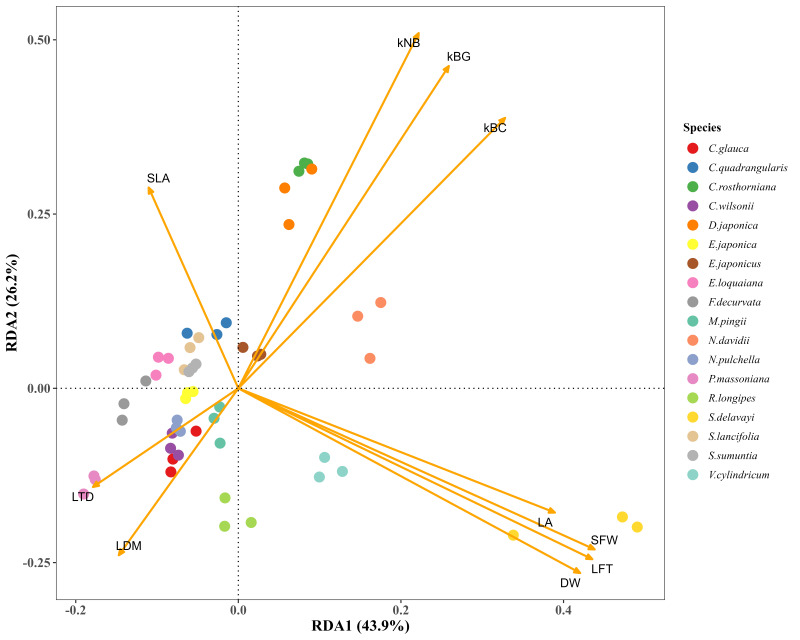
RDA analysis of leaf functional traits and K-values across different species. Saturated Fresh Weight, SFW (g); Dry Weight, DW (g); Thickness of leaf, LFT (mm/single); leaf area, LA (cm^2^/single); Leaf Dry Matter Content, LDM (g/g); Specific leaf area, SLA (cm^2^/g); Leaf tissue density, LTD (g/cm^3^); *k*BC, decomposition rate in BC habitat; *k*BG, decomposition rate in BG habitat; *k*NB, decomposition rate in NB habitat.

Evaluating the impact of litter decomposition drivers across distinct habitats requires studying decomposition at comparable species levels. Our study conducted a multi-species decomposition experiment across distinct leaf functional traits, demonstrating that the litter decomposition rate is closely related to litter traits (Table 2 and Figure 5).

In this study, the decomposition periods of *Cyclobalanopsis glauca*, *C. wilsonii*, *N. pulchella* and *L. elongata* were notably prolonged, even exceeding the turnover durations of certain species (e.g., *Camellia rosthorniana* and *D. japonica*) by multiple folds. Bamboo leaf litter consistently exhibited longer decomposition half-time (t_0.5_) compared to tree leaf litter (Table S3), a phenomenon potentially linked to silicon accumulation in bamboo phytoliths that mechanically inhibits microbial colonization and enzymatic hydrolysis [28]. Compared to ground habitats, species such as *P. massoniana*, *S. lancifolia*, *S. setchuensis*, *L. elongata* exhibited the slowest decomposition rates in the bamboo canopy. These species possess leathery or needle-like leaves with thick cuticles. Coriaceous leaves resist decomposition through lignified vascular bundles and phenolic compounds [44], while waxy cuticles and recalcitrant lipid compounds in coniferous species create biochemical barriers to decay. Furthermore, being suspended in the bamboo canopy, these litter types are less exposed to detritivore fragmentation and leaching, further delaying decomposition. In contrast, the leaves of *C. wilsonii* became brittle upon air-drying and were more susceptible to physical fragmentation (e.g., by wind or bamboo canopy movement), leading to faster decomposition in the bamboo canopy than on the ground. Key functional traits, including leaf tissue density (LTD) and leaf dry matter content (LDMC), emerged as significant negative predictors of decomposition rates [45]. Among other species, those with membranous leaves decomposed the most rapidly across all three habitats, followed by those with leathery leaves. This hierarchy reflects structural and chemical controls: due to limited structural defenses, membranous leaves undergo rapid leaching and microbial fragmentation [44].

### 4.5. Limitations and Future Research

The conclusions of this study are derived from a single forest type, and their extrapolation to other ecosystems requires careful assessment. As key environmental factors (e.g., temperature, humidity) and biological factors (e.g., soil microbial and faunal communities) were not systematically monitored, the specific mechanisms by which the bamboo canopy influences litter decomposition remain to be fully elucidated. Future research should be expanded to diverse ecological regions and incorporate synchronized monitoring of multiple factors to clarify the underlying pathways. From a management perspective, dense understory bamboo layers significantly alter the distribution pattern and decomposition process of canopy litter. It is therefore recommended that bamboo density regulation be incorporated into nutrient cycle management strategies during subtropical mountain forest cultivation, so as to maintain soil ecological function.

## 5. Conclusions

Understory dwarf bamboo effectively intercepts canopy litterfall, thereby reducing the amount of litter that reaches the soil surface. Compared to the falling ground surface litter, intercepted litter exhibits slower decomposition efficiency and a lower rate of carbon release. However, the nutrient release rate of potassium in the bamboo canopy habitat was significantly higher than that in ground surface habitats. Furthermore, we found that litter with leathery or needle leaves decomposes more slowly in the bamboo canopy environment. Overall, the understory dwarf bamboo reduces nutrient cycling efficiency by intercepting litter, thereby altering its spatial distribution and delaying the decomposition of intercepted litter. We propose that the understory dwarf bamboo plays a non-negligible role in altering the drivers of nutrient cycling of litter in forest ecosystems.

## Figures and Tables

**Table 1:** Nutrient release rates of litter leaves in different decomposition habitats. BC represents bamboo-canopy habitat; BG represents bamboo-ground habitat; NB represents non-bamboo habitat.

Nutrient Release Rates (%)	Decomposition Time (d)	BC	BG	NB
C	90	47.148 ± 1.000 a	48.808 ± 1.080 a	46.551 ± 0.972 a
	180	54.586 ± 1.005 b	58.863 ± 1.234 a	53.449 ± 1.119 b
	270	57.763 ± 1.052 b	63.564 ± 1.229 a	58.946 ± 1.259 b
	360	60.184 ± 1.108 b	67.831 ± 1.246 a	62.534 ± 1.252 b
N	90	−45.641 ± 3.447 a	−49.034 ± 3.848 a	−46.553 ± 3.295 a
	180	−52.450 ± 3.539 a	−59.682 ± 3.620 a	−51.813 ± 3.301 a
	270	−47.162 ± 3.031 a	−43.911 ± 3.04 a	−43.830 ± 3.074 a
	360	−44.592 ± 3.121 a	−43.776 ± 3.417 a	−42.562 ± 3.058 a
P	90	−11.491 ± 3.894 a	−37.128 ± 4.519 b	−36.979 ± 4.789 b
	180	−41.122 ± 5.202 a	−43.061 ± 5.631 a	−34.042 ± 4.876 a
	270	−27.086 ± 4.937 a	−14.387 ± 4.425 a	−25.328 ± 4.677 a
	360	−6.967 ± 4.071 a	−16.454 ± 5.080 a	−8.598 ± 3.899 a
K	90	86.302 ± 0.483 a	83.340 ± 0.667 b	84.591 ± 0.581 b
	180	83.503 ± 0.549 a	72.895 ± 1.023 c	77.996 ± 1.113 b
	270	84.466 ± 0.697 a	84.757 ± 0.442 a	81.897 ± 0.767 b
	360	86.393 ± 0.456 a	83.019 ± 0.724 b	83.154 ± 0.629 b
C/N	90	9.495 ± 0.353 a	9.478 ± 0.395 a	9.740 ± 0.403 a
	180	7.893 ± 0.291 a	6.959 ± 0.326 b	8.266 ± 0.338 a
	270	7.522 ± 0.309 a	6.864 ± 0.320 a	7.549 ± 0.350 a
	360	7.237 ± 0.302 a	5.909 ± 0.288 b	7.024 ± 0.349 a
N/P	90	25.653 ± 0.874 a	20.827 ± 0.577 b	20.708 ± 0.529 b
	180	21.025 ± 0.656 a	21.101 ± 0.634 a	21.645 ± 0.839 a
	270	23.219 ± 0.815 a	25.144 ± 1.348 a	22.484 ± 0.646 a
	360	26.546 ± 0.749 a	24.667 ± 0.983 a	25.139 ± 0.663 a

Note: Nutrient release rate (%) = [(initial nutrient content − remaining nutrient content at retrieval)/initial nutrient content] × 100. Lowercase letters indicate significant differences in nutrient release rates among different decomposition environments at the same decomposition time, *p* < 0.05.

**Table 2:** Repeated-measures ANOVA results for the residual mass and contents of C, N, P, K, C/N and N/P.

Source of Variation	F-Value						
	MR	C	N	P	K	C/N	N/P
Species (SP)	205.05 ***	499.85 ***	150.31 ***	5.09 ***	19.69 ***	889.98 ***	127.92 ***
Habitat (H)	148.31 ***	78.48 ***	0.28 ^ns^	2.41 ^ns^	8.38 ***	213.45 ***	25.93 ***
Time (T)	12,078.53 ***	344.34 ***	917.61 ***	6.99 **	1637.87 ***	2307.08 ***	55.09 ***
SP × H	7.23 ***	4.23 ***	6.62 ***	1.04 ^ns^	0.815 ^ns^	57.06 ***	8.51 ***
SP × T	15.52 ***	9.95 ***	10.99 ***	1.44 ^ns^	14.71 ***	20.64 ***	2.99 ***
H × T	30.06 ***	17.21 ***	5.22 ***	0.61 ^ns^	1.576 ^ns^	49.78 ***	15.73 ***
SP × H × T	1.94 ***	2.83 ***	2.63 ***	1.02 ^ns^	0.86 ^ns^	10.73 ***	3.01 ***

Note: MR (Residual mass). ***, *p* < 0.001. **, *p* < 0.01. ns, *p* > 0.05.

## Data Availability

The original contributions presented in this study are included in the article/Supplementary Material. Further inquiries can be directed to the corresponding author.

## Supplementary Materials

The following supporting information can be downloaded at: https://www.mdpi.com/article/10.3390/plants14203135/s1. Table S1. Species information of dominant trees in the 1-hectare permanent plot. Table S2. Species Information for Decomposition Experiments. Table S3. Exponential regression equations for the residual rate of fallen leaf mass over time, half-life, and turnover time for decomposition.
