# Supplementary material for: Understory Dwarf Bamboo Modulates Leaf Litter Decomposition via Interception-Induced Litter Redistribution and Space-Dependent Decomposition Dynamics: A Case Study from Jinfo Mountain, China
Source: Plants (Basel). 2025 Oct 11;14(20):3135. doi: 10.3390/plants14203135 (PMC12566774; doi:10.3390/plants14203135)
Supplement: Supplementary file 1 [file plants-14-03135-s001.zip › plants-3879075-supplementary.pdf]

Supplementary Tables

Table S1 Species information of dominant trees in the 1-hectare permanent plot

Species	*n*	Mean height (m)	Mean under branch height (m)	Mean Diameter at Breast Height (cm)	Mean crown width (m × m)
Symplocos sumuntia	89	9.4 ± 4.5	4.6 ± 2.7	11.4 ± 6.3	3.0×3.1
Cinnamomum wilsonii	65	8.6 ± 3.4	4.8 ± 2.6	11.0 ± 5.8	2.6×2.7
Symplocos setchuensis	45	8.5 ± 3.2	5.0 ± 2.4	10.2 ± 4.7	2.5×2.6
Symplocos lancifolia	37	8.7 ± 3.6	5.1 ± 2.5	10.5 ± 5.2	2.6×2.8
Nothopanax davidii	36	8.1 ± 3.0	4.2 ± 2.1	12.3 ± 5.9	4.8×3.1
Neolitsea pulchella	34	9.8 ± 3.9	5.2 ± 2.6	12.8 ± 6.5	3.2×3.7
Lithocarpus hancei	33	9.5 ± 4.2	5.0 ± 2.8	13.1 ± 7.0	2.8×3.0
Symplocos anomala	32	8.3 ± 3.5	4.9 ± 2.7	11.9 ± 6.4	2.5×2.7
Litsea elongata	30	8.7 ± 4.0	4.6 ± 2.5	11.5 ± 5.6	4.6×2.9
Viburnum setigerum	25	7.8 ± 2.8	4.1 ± 2.2	9.1 ± 3.9	2.4×2.6
Rhododendron longipes	24	10.1 ± 3.6	5.3 ± 2.4	10.9 ± 4.8	2.7×2.9
Eurya japonica	23	9.1 ± 3.9	5.6 ± 2.7	10.2 ± 5.0	2.9×3.4
Rhododendron simsii	22	8.6 ± 3.8	4.4 ± 2.6	11.8 ± 6.2	2.5×2.8
Fraxinus insularis	21	9.7 ± 4.5	6.1 ± 3.2	9.8 ± 4.6	2.6×3.0
Dendrobenthamia japonica	20	11.2 ± 4.0	6.4 ± 2.8	11.9 ± 4.5	3.1×3.5
Photinia beauverdiana	19	8.1 ± 3.2	5.5 ± 2.4	13.2 ± 6.8	2.7×3.1
Ilex chinensis	18	8.2 ± 3.5	4.6 ± 2.3	9.5 ± 4.2	2.5×2.9
Camellia japonica	18	8.9 ± 4.2	4.1 ± 2.3	13.1 ± 7.8	2.8×3.2
Carpinus viminea	17	9.3 ± 4.0	5.2 ± 2.6	10.4 ± 4.7	2.7×3.0
Machilus pingii	17	6.9 ± 0.9	4.0 ± 0.9	6.4 ± 0.7	2.3×2.5
Cyclobalanopsis glauca	17	11.1 ± 3.5	4.0 ± 1.8	11.8 ± 3.0	2.8×3.0
Elaeocarpus japonicus	17	12.5 ± 0.0	4.5 ± 0.0	16.5 ± 0.0	4.0×3.0

Symplocos theophrastifolia	16	9.8 ± 4.2	5.1 ± 2.5	11.6 ± 5.9	3.0×3.3
Viburnum cylindricum	16	7.1 ± 1.8	4.0 ± 2.8	4.9 ± 0.1	2.3×2.5
Eurya loquaiana	15	12.8 ± 0.0	3.8 ± 0.0	15.9 ± 0.0	6.0×7.0
Camellia rosthorniana	15	7.1 ± 1.8	4.0 ± 2.8	4.9 ± 0.1	2.3×2.5
Pinus massoniana	14	13.2 ± 4.8	7.5 ± 2.5	22.4 ± 7.5	5.8×4.7
Lyonia ovalifolia	11	7.3 ± 1.8	5.8 ± 0.8	9.1 ± 1.8	2.3×4.9
Schefflera delavayi	10	8.4 ± 1.8	4.9 ± 2.3	8.1 ± 2.3	2.3×2.4

Note： *n* indicates the number of individuals of the species in the 1-hectare plot. Data are presented as mean ± standard deviation (SD).

Table S2 Species Information for Decomposition Experiments

Shorthand	Species	SFW (g)	DW (g)	LFT (mm)	LA (cm^2/single)	LDM (g/g)	SLA (cm^2/g)	LTD (g/cm^3)
C. rosthorniana	*Camellia rosthorniana*	0.403±0.011	0.126±0.005	0.265±0.008	16.745±0.564	0.312±0.004	133.102±4.179	2.850±0.068
C. wilsonii	*Cinnamomum wilsonii*	0.351±0.024	0.176±0.019	0.263±0.021	17.271±1.601	0.499±0.022	98.515±2.403	3.897±0.232
C. quadrangularis	*Chimonobambusa quadrangularis*	0.338±0.036	0.185±0.02	0.262±0.026	31.404±1.915	0.549±0.038	171.935±10.962	2.259±0.097
S. lancifolia	*Symplocos lancifolia*	0.240±0.016	0.102±0.009	0.171±0.011	11.308±1.177	0.426±0.035	110.588±1.518	5.341±0.415
L. elongata	*Litsea elongata* var. *subverticillata*	0.474±0.082	0.304±0.034	0.389±0.056	41.389±3.858	0.663±0.078	142.304±27.094	1.934±0.163
E. japonica	*Eurya japonica*	0.316±0.011	0.119±0.004	0.217±0.007	10.332±0.434	0.376±0.003	87.014±1.050	5.305±0.230
N. pulchella	*Neolitsea pulchella*	0.359±0.016	0.173±0.007	0.266±0.012	17.930±0.663	0.483±0.002	103.692±1.588	3.643±0.145
P. massoniana	*Pinus massoniana*	0.027±0.001	0.013±0.001	0.020±0.001	0.798±0.032	0.458±0.015	63.107±0.771	79.698±3.925
C. glauca	*Cyclobalanopsis glauca*	0.424±0.045	0.261±0.006	0.342±0.023	32.520±0.536	0.629±0.062	124.667±1.934	2.3680±0.175
M. pingii	*Machilus pingii*	0.646±0.039	0.258±0.018	0.452±0.028	24.047±0.342	0.400±0.006	93.795±4.994	2.377±0.026
E. japonicus	*Elacocarpus japonicus*	0.634±0.048	0.273±0.027	0.453±0.037	24.951±3.067	0.430±0.013	91.048±3.977	2.474±0.282
S. sumuntia	*Symplocos sumuntia*	0.325±0.013	0.111±0.003	0.218±0.008	12.563±0.541	0.343±0.005	112.74±2.322	4.087±0.221
V. cylindricum	*Viburnum cylindricum*	1.529±0.169	0.711±0.023	1.120±0.095	71.140±2.201	0.473±0.036	100.109±1.983	0.903±0.062
S. delavayi	*Schefflera delavayi*	4.069±0.357	1.609±0.086	2.839±0.139	99.720±41.855	0.406±0.061	64.911±27.970	1.680±1.318
D. japonica	*Dendrobenthamia japonica*	0.430±0.044	0.161±0.018	0.296±0.031	18.977±1.765	0.374±0.005	118.253±2.767	2.915±0.263
E. loquaiana	*Eurya loquaiana*	0.169±0.009	0.059±0.003	0.114±0.006	8.703±0.224	0.351±0.002	147.027±3.598	5.991±0.177
N. davidii	*Nothopanax davidii*	1.356±0.075	0.545±0.028	0.950±0.047	67.605±3.321	0.403±0.020	124.202±2.549	0.853±0.054
F. decurvata	*Fargesia decurvata*	0.140±0.019	0.084±0.003	0.112±0.009	15.059±0.193	0.620±0.091	180.449±5.539	5.040±0.446

Note: Saturated Fresh Weight, SFW (g); Dry Weight, DW (g); Thickness of leaf, LFT (mm/single); leaf area, LA (cm^2/single); Leaf Dry Matter Content, LDM (g/g); Specific leaf area, SLA (cm^2/g); Leaf tissue density, LTD (g/cm^3)

Table S3 Exponential regression equations for the residual rate of fallen leaf mass over time, half-life, and turnover time for decomposition.

Species	Habitat	Regression equation	Decomposition constant (k)	R^2	Half-life (y)	Turnover (y)
Camellia rosthorniana	BC	$y=93.05e^{-0.58t}$	0.58	0.77^{**}	1.20	5.19
	BG	$y=96.90e^{-0.78t}$	0.78	0.89^{**}	0.89	3.86
	NB	$y=96.43e^{-0.72t}$	0.72	0.89^{**}	0.96	4.17
Cinnamomum wilsonii	BC	$y=90.49e^{-0.29t}$	0.29	0.77^{**}	2.35	10.17
	BG	$y=90.71e^{-0.22t}$	0.22	0.78^{**}	3.20	13.83
	NB	$y=91.72e^{-0.2t}$	0.20	0.83^{**}	3.50	15.13
Chimonobambusa quadrangularis	BC	$y=89.74e^{-0.31t}$	0.31	0.71^{**}	2.24	9.66
	BG	$y=91.55e^{-0.38t}$	0.38	0.81^{**}	1.82	7.86
	NB	$y=90.05e^{-0.33t}$	0.33	0.77^{**}	2.12	9.17
Symplocos lancifolia	BC	$y=91.46e^{-0.29t}$	0.29	0.80^{**}	2.38	10.29
	BG	$y=92.89e^{-0.46t}$	0.46	0.83^{**}	1.51	6.54
	NB	$y=92.89e^{-0.46t}$	0.46	0.88^{**}	1.51	6.54
Litsea elongata var. subverticillata	BC	$y=89.66e^{-0.17t}$	0.17	0.71^{*}	3.98	17.21
	BG	$y=94.01e^{-0.24t}$	0.24	0.92^{**}	2.83	12.24
	NB	$y=89.89e^{-0.16t}$	0.16	0.72^{*}	4.25	18.38
Eurya japonica	BC	$y=90.58e^{-0.3t}$	0.30	0.80^{**}	2.30	9.94
	BG	$y=89.66e^{-0.26t}$	0.26	0.76^{**}	2.67	11.56
	NB	$y=89.51e^{-0.22t}$	0.22	0.74^{*}	3.15	13.63
Neolitsea pulchella	BC	$y=89.55e^{-0.23t}$	0.23	0.73^{*}	2.97	12.81
	BG	$y=92e^{-0.26t}$	0.26	0.84^{**}	2.65	11.45
	NB	$y=90.86e^{-0.26t}$	0.26	0.79^{**}	2.69	11.63
Pinus massoniana	BC	$y=90.82e^{-0.18t}$	0.18	0.75^{**}	3.76	16.27
	BG	$y=91.68e^{-0.24t}$	0.24	0.84^{**}	2.92	12.62
	NB	$y=90.71e^{-0.21t}$	0.21	0.77^{**}	3.33	14.40
Cyclobalanopsis glauca	BC	$y=90.66e^{-0.25t}$	0.25	0.79^{**}	2.78	12.00
	BG	$y=92.78e^{-0.29t}$	0.29	0.89^{**}	2.39	10.34
	NB	$y=90.18e^{-0.23t}$	0.23	0.77^{**}	3.01	13.02
Machilus pingii	BC	$y=88.75e^{-0.23t}$	0.23	0.69^{*}	2.97	12.84
	BG	$y=88.75e^{-0.23t}$	0.23	0.93^{**}	2.97	12.84
	NB	$y=93.75e^{-0.33t}$	0.33	0.93^{**}	2.12	9.16
Elacocarpus japonicus	BC	$y=90.85e^{-0.32t}$	0.32	0.77^{**}	2.16	9.35
	BG	$y=93.13e^{-0.49t}$	0.49	0.80^{**}	1.43	6.17
	NB	$y=90.74e^{-0.35t}$	0.35	0.81^{**}	2.00	8.64
Symplocos sumuntia	BC	$y=88.01e^{-0.23t}$	0.23	0.65^{*}	2.97	12.82
	BG	$y=90.48e^{-0.29t}$	0.29	0.80^{**}	2.38	10.29
	NB	$y=90.19e^{-0.26t}$	0.26	0.78^{**}	2.65	11.45
Viburnum cylindricum	BC	$y=91.45e^{-0.3t}$	0.30	0.83^{**}	2.28	9.87
	BG	$y=91.45e^{-0.3t}$	0.30	0.83^{**}	2.28	9.87

	NB	$y=91.53e^{-0.31t}$	0.31	0.84^{**}	2.22	9.58
	BC	$y=90.78e^{-0.44t}$	0.44	0.72^{*}	1.58	6.82
Schefflera delavayi	BG	$y=91.29e^{-0.41t}$	0.41	0.74^{*}	1.69	7.29
	NB	$y=93.45e^{-0.41t}$	0.41	0.84^{**}	1.68	7.24
	BC	$y=94.26e^{-0.56t}$	0.56	0.84^{**}	1.23	5.33
Dendrobenthamia japonica	BG	$y=97.92e^{-0.9t}$	0.90	0.89^{**}	0.77	3.33
	NB	$y=96.5e^{-0.74t}$	0.74	0.88^{**}	0.94	4.06
	BC	$y=89.94e^{-0.26t}$	0.26	0.74^{*}	2.70	11.67
Eurya loquaiana	BG	$y=90.69e^{-0.28t}$	0.28	0.80^{**}	2.50	10.80
	NB	$y=90.5e^{-0.23t}$	0.23	0.81^{**}	2.99	12.92
	BC	$y=91.71e^{-0.49t}$	0.49	0.77^{**}	1.43	6.17
Nothopanax davidii	BG	$y=93.96e^{-0.52t}$	0.52	0.91^{**}	1.34	5.81
	NB	$y=91.85e^{-0.43t}$	0.43	0.82^{**}	1.60	6.91
	BC	$y=90.12e^{-0.24t}$	0.24	0.76^{**}	2.93	12.67
Fargesia decurvata	BG	$y=92.49e^{-0.27t}$	0.27	0.88^{**}	2.58	11.15
	NB	$y=90.26e^{-0.24t}$	0.24	0.79^{**}	2.89	12.47
	BC	$y=90.1e^{-0.28t}$	0.28	0.76^{**}	2.46	10.65
Species level	BG	$y=92.1e^{-0.35t}$	0.35	0.85^{**}	1.97	8.52
	NB	$y=91.06e^{-0.29t}$	0.29	0.82^{**}	2.37	10.23

Note: ** denotes that the model has passed the significance test ($P < 0.01$); * denotes that the model has passed the significance test ($P < 0.05$). BC represents the bamboo canopy habitat, BG refers to the bamboo-covered ground, and NB denotes the bamboo-free ground. Species level: the average value of litter decomposition of 18 species.
